# Autotransplantation of mature impacted tooth to a fresh molar socket using a 3D replica and guided bone regeneration: two years retrospective case series

**DOI:** 10.1186/s12903-019-0945-8

**Published:** 2019-11-14

**Authors:** Ye Wu, Jiaming Chen, Fuping Xie, Huanhuan Liu, Gang Niu, Lin Zhou

**Affiliations:** 1 Department of Oral and Maxillofacial Surgery, School and Hospital of Stomatology, Fujian Medical University Fujian Stomatological Hospital, Fuzhou, Fujian China; 2 Department of Oral Implantology, School and Hospital of Stomatology, Fujian Medical University Fujian Stomatological Hospital, Fuzhou, Fujian China

**Keywords:** Tooth autotransplantation, Mature impacted tooth, 3D replica model, Guided bone regeneration

## Abstract

**Background:**

The aim of this study was to evaluate the clinical outcome of autotransplantation of mature third molars to fresh molar extraction sockets using 3D replicas.

**Methods:**

Ten patients underwent teeth autotransplantation with or without GBR. We observed the mobility, percussion, radiography examination, the probing depth and the masticatory function of the transplanted teeth during 2 years following up, which were transplanted into fresh molar sockets by using 3D replicas, and GBR when it is necessary.

**Results:**

The average extra-oral time of donor tooth had been shortened to 1.65 min when used the 3D replica. Some probing depth of the transplanted tooth were deeper than 3 mm at 4 or 5 weeks temporarily. And one patient felt slight sensitive when chewing with soft food at 4 weeks, then disappeared. The clinical examination of the autotransplantation teeth during 1 year follow-up showed no sign of failure.

**Conclusions:**

The tooth autotransplantation using 3D replica with or without GBR is an effective method which can reduce the extra-oral time of the donor teeth and may result in less failure.

## Background

The tooth autotransplantation is a predictable method to replace a tooth that needs to be extracted due to caries, trauma, or tooth fracture. Since it was first introduced by Fauchard in his book, Le Chirurgien Dentiste, in 1728, the clinical protocol had been developed for hundreds of years [1–3]. Its brief process is that the donor tooth (mostly an impact tooth or a supernumerary tooth) is extracted for the insertion of a prepared recipient socket [4]. Compared to dental implant, the tooth autotransplantation is a better way to restore missing teeth for its proprioception, the vital periodontium, preservation of alveolar bone volume and the papilla [5], and also better than a fixed bridge.

Many previous studies have demonstrated that third molars, premolars, impacted teeth and supernumerary teeth can be a donor tooth in the clinical practice [6–8]. The incidence of the extraction of the compromised molars is much higher than in other teeth, especially in young Chinese range from 25 to 30 years old. The transplantation of a third molar to replace compromised first or second molar has more practical value. The survival rates of tooth autotransplantation with incomplete root formation after 1, 5 and 10 years were 97.4, 97.8 and 96.3% respectively [9]. However, some studies showed that the estimated 10-years success rate of a transplanted premolar with mature root was 81.6% which is much higher than that of a molar, with a 33.8% 10-years success rate [10]. Many factors affect the success of tooth autotransplantation, such as the stage of root development, surgical trauma, the recipient site (local inflammation, alveolar bone volume and quality), the surgery procedure (stabilization method, use of intraoperative drugs and storage) [11–13]. The lower success and survival rates of the transplanted molar can be related to more complex root anatomy, more tissue trauma during extraction, and the requirement of high individual surgical skill [14]. The most important factor that affect a successful tooth autotransplantation is the preservation of the healthy periodontal tissue [15]. But the duration of the extra-oral time and the try-ins into the recipient socket will damage the periodontal tissues of the donor teeth.

Researchers are constantly exploring how to shorten the extraoral time of donor tooth, reduce the damage to the periodontal tissue, and improve the surgeon’s skill. There are many methods attempting to reduce the extraoral time of donor tooth [16]. With the development of radiography and 3D printing technology, a precisely replica of donor teeth can be fabricated by a 3D printer, according to the data of cone beam computed tomography of the donor tooth. Many case reports have indicated that the use of a 3D replica of donor tooth can decrease the extraoral time and increase the ease of surgery [17]. Lee and Shahbazian have used computer-aided rapid prototyping for tooth transplantation and shorten the extraoral time [18, 19]. EzEldeen et al. reported that the CBCT-guided tooth autotransplantation could improve the survival rate to 92% compared with conventional way, and 3D analysis can provide insights into the patterns of healing [20].

The bone defect between the prepared socket and the donor tooth is inevitable when transplantation was done in a larger fresh extraction socket. And the bone graft materials were need to fill the bone defect. Yu et al. have autotransplanted canine combined with guided bone regeneration, which show an acceptable result during 7.1 years following up [21]. The technique of guided bone regeneration had been widely used in the implantation. Yu. et al. also reported that the survival rate of the autotransplantation of third molars with completely formed roots in both surgically created and fresh extraction sockets were 93.1 and 95.2% during 10 years following up [22]. However, the investigations of the clinical outcomes and the survival rate of tooth autotransplantation to a fresh extraction socket using 3D replica and association with GBR are still lacking.

Therefore, the purpose of this study is to evaluate the clinical outcomes of the transplantation of third molar to fresh first or second molar extraction sockets by using the 3D replica of donor teeth and grafting with autogenous bone to fill the gap between the tooth and the prepared socket when necessary.

## Methods

### Study population and design

This was a retrospective observational study of autotransplantation of third molars into fresh first or second molar extraction sockets simultaneously using a 3D replica and grafting with autogenous bone mixed with concentrated growth factors (CGFs) in 10 patients (8 males and 2 females) (ages ranging from 19 to 42 years) between September 2016 and August 2017. All the patients were consecutively collected from Department of Oral and Maxillofacial Surgery, Affiliated Stomatological Hospital of Fujian Medical University. All patients were informed about the surgical treatment procedure. The study design was performed in accordance with the Helsinki Declaration (revised in 2008).

The patients included in this study reach the following criteria:

1. First or second molar need to be extracted.
2. Third molar with mature root need to be extracted.
3. Recipient site without local acute inflammatory.
4. The rest bone height of the recipient site is enough for the donor tooth (the height from alveolar ridge crest to inferior alveolar nerve).
5. Systemic diseases such as diabetes mellitus and hypertension, which is not suit for oral surgery, were absent.

### Preoperative work-up

**Fig. 1:**
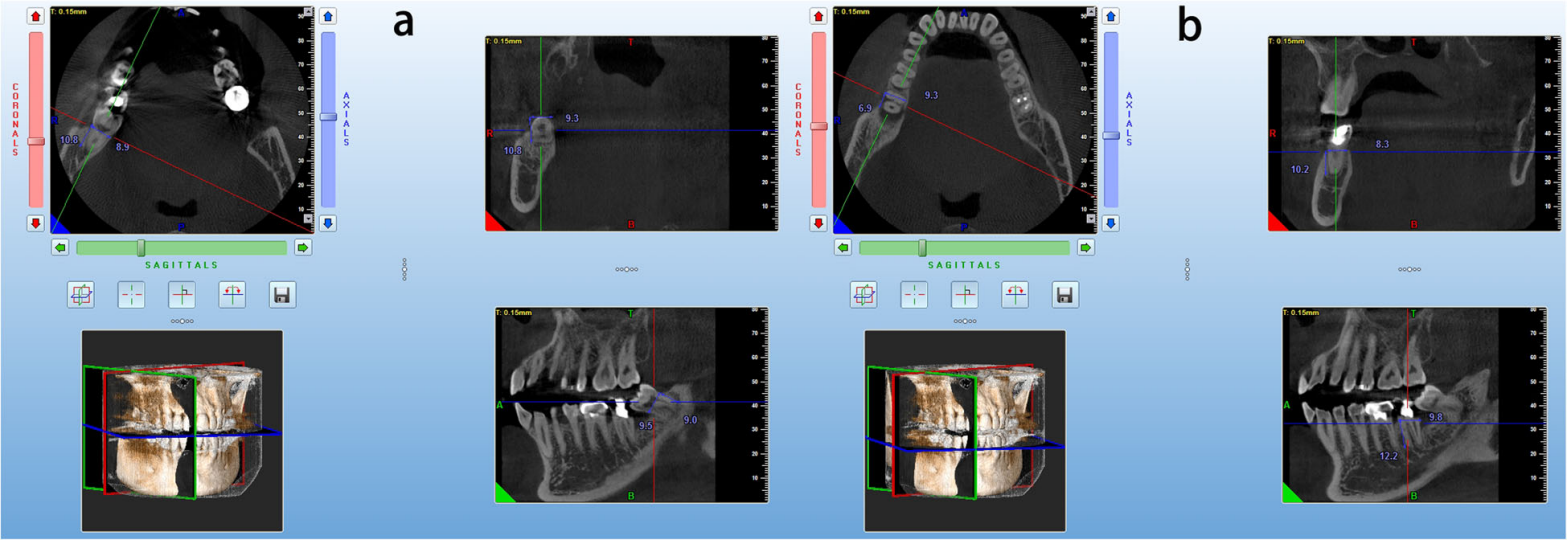
The measurements of the CBCT for preoperative evaluation. **a** the measurements of the mesio-distal and bucco-lingual dimension and height of the donor tooth at three different axes. **b** the measurements of the mesio-distal and bucco-lingual dimension and height of the recipient site at three different axes

All patients underwent a low-dose CBCT imaging using the NewTom GiANO (NewTom, Italy) with voxel size 0.150 mm, tube voltage of 90 kV, current of 7.00 mA, and exposure time of 9 s. All patients received a cone-beam computed tomography (CBCT) examination to analysis the compromised tooth and the donor tooth (the stage of the root development and the shape of the root), then the mesio-distal, bucco-lingual dimension, height of the donor tooth and the recipient site were measured to evaluate the adaptability of the donor tooth to the fresh extraction socket as it showed in Fig. 1.

After comparing the root of the donor tooth with the extracted tooth, we can know that whether the extraction socket need to be prepared or GBR it needed to filled the gap between the donor tooth and the extraction socket. For patients with horizontal alveolar bone or buccal bone loss at the recipient site, GBR would also need performed. And if GBR is needed, the concentrated growth factors (CGFs) would be prepared before surgery. Whole blood drew from the patient was centrifuged using a tabletop centrifuge (Medifuge, Silfradenstsr, S. Sofia, Italy) and it was divided into four layers as described by Bozkurt et al. The CGF layer, which was the second growth factor and stem cell layer of the four layers, was separated using sterile scissor.

**Fig. 2:**
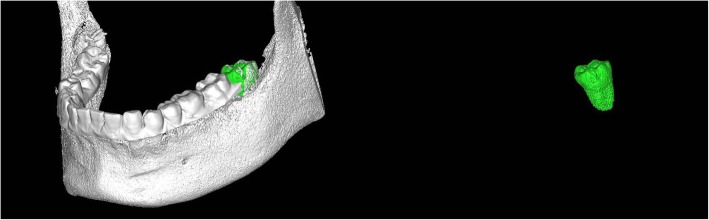
The segmentation of the donor tooth from mandible in the Materialize Proplan software

The CBCT data was imported into Materialize Proplan software, this allowed the segmentation of the donor tooth as it show in Fig. 2. Then 3D replica of the donor teeth, made of resin material, was fabricated by a 3D printer (Vida, Envision TEC) according to the segmentation data from the CBCT. All patients underwent an overall dental hygiene assessment, teeth washing or scaling, and root planning 1 week before surgery, if necessary.

### Surgical proceduce

All the surgical proceduces were performed by the same surgeon, who had more than 20 years of experience in oral surgery. Block anesthesia of the inferior alveolar nerve was performed when the donor teeth and the recipient site were in the mandibular; local anesthesia was performed when the donor teeth or the recipient site were in the maxillary. Local anesthesia was achieved with articaine chlorhydrate 4% and adrenaline 1:100000.

**Fig. 3:**
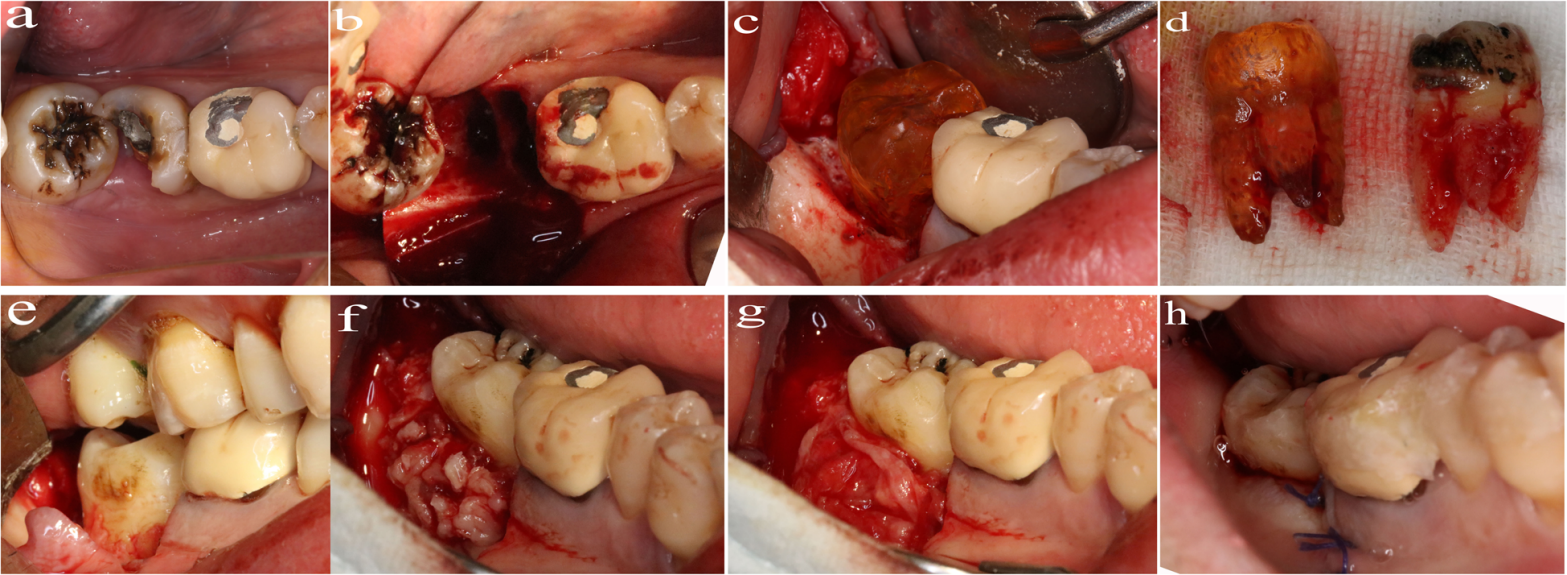
The surgical proceduce of autotransplantation of a mature third molar tooth in a fresh socket of second molar tooth: **a** compromised second molar tooth. **b** fresh socket of the second molar tooth after extraction. **c** try-in of the 3D replica of the donor tooth. **d** the 3D replica was almost the same of the donor maxillary third tooth. **e** try-in of the donor tooth. **f** grafting of the autogenous bone in the buccul and distal side of the donor tooth. **g** covering with CGF membrane. **h** suturing the flap and fixed the autotransplantation tooth

A crevicular incision was made from second premolar to third molar, and the vertical releasing incision in distal side was made if necessary. The compromised molar was extracted by minimally invasive maneuver, using high-speed fissure bur (SINOL) and a dental elevator or forceps (Stoma). The preparation of recipient site was done by piezosurgery according the root shape of the 3D replica of the donor teeth, which was sterilized by ethylene oxide before surgery. For the patients who need performed GBR, which was derived from the CBCT analysis before surgery, the bone fragment was collected during the preparation process. The impacted tooth was also extracted by minimally invasive technique, using a dental elevator or forceps (Stoma). We put the donor teeth into the recipient socket and checked whether it achieve an optimal fit. If there was bone defect around the donor tooth or the fresh extraction socket was larger than the root of the donor tooth, we grafted the autogenous bone to fill the gaps or the bone defects. Then the bone graft area was covered by CGF membrane which was done before surgery. Finally, the flap was repositioned and sutured. The transplanted teeth were stabilized with splints attached to the adjacent teeth which were carried out with a multi-layer fiber-glass band. The brief surgical procedure of the tooth autotransplantation was showed in Fig. 3.

### Postoperative treatment

After surgery, all the patients received mouth rinsing for 1 week. After 1 week, the sutures were removed and the wound was cleaned by normal saline. The preparation of the root canal was performed 2 weeks after surgery and the filling of the root canal was done 5 weeks after surgery. The multi-layer fiber-glass band was removed 5 weeks after surgery.

### Postoperative examination

Follow-up recalls were scheduled for1, 2, 4 weeks and for 3, 6,12, 24 months. At each time of the follow-up the mobility and percussion were checked, while the probing depth of the mesial-buccal, buccal, distal-buccal, mesial lingual, lingual, and distal-lingual of the transplantated teeth and the masticatory function were checked 1, 3, 6, 12 and 24 months after surgery. The radiography examination was taken before surgery and immediate, 1, 3, 12, 24 months after surgery. We defined the normal masticatory function as patients’ ability to chew normal food without pain or discomfort. The primary success criteria of the transplanted tooth were followed according to the described by Tsukiboshi [23]. In terms of the radiography (1) normal space of the parodontium; (2) no sign of progressivity absorb of the root; (3) the exist of the lamina dura. In terms of clinical examination (1) normal mobility; (2) normal percussion sound; (3) no periodontal pocket; (4) no sign of inflammation; (5) no discomfortable; (6) normal function of chewing.

## Results

Retrospective, we evaluated 10 patients (8 male and 2 females, mean age 31.6 + 8.75, range from 19 to 42 years) who underwent transplantation of their third molar to their fresh first or second molar extraction socket, using a 3D replica. Transplantation of the mature impacted tooth to compromised molar which was in the same region occurred in six cases, the other were in different region. The basic information of the patients is recorded Table 1.

**Table 1:** Basic characteristics of the patients

No.	Age ranges	Donor tooth	Recipient site	Reason for extraction	Extrao-ral time (min)	GBR (autogenous bone)
1	30–40	48	46	Root fracture	4.0	Y
2	20–30	48	46	Root fracture	Less than 1 min	N
3	20–30	38	36	Severe caries	Less than 1 min	N
4	20–30	38	16	Furcation involvement	Less than 1 min	Y
5	40–50	28	47	Severe caries	3.5	Y
6	30–40	28	37	Severe caries	1.0	Y
7	30–40	48	47	Root fracture	1.0	Y
8	30–40	48	47	Severe caries	1.5	Y
9	40–50	28	37	Severe caries	1.5	Y
10	10–20	38	37	Severe caries	1.0	Y

### Preoperative evaluation

The CBCT measurements and the Materialize Proplan software allowed the surgeon to evaluate which mature impacted tooth is best donor tooth to the recipient extraction socket beforehand according to the mesio-distal and bucco-lingual dimension and height of the donor tooth and the recipient site and the number and shape of the root, and whether the GBR is needed. The site of the donor tooth, recipient site, reason for extraction, and whether guided bone regeneration (GBR) was performed are also showed in Table 1.

### Surgical procedure

The 3D replica model was used as a guide for preparing the fresh extraction molar socket. When the 3D replica was best fit for the prepared socket, we started to extract the donor tooth. The extraoral time of each case were showed in Table 1. The average extraoral time of the donor teeth spent was 1.35 min, and three donor teeth were transplanted in the recipient socket less than 1 min after extraction. But there were two cases cost 3.5 and 4 min respectively due to the error range of the 3D replica.

### Clinical and radiographic evaluation

**Fig. 4:**
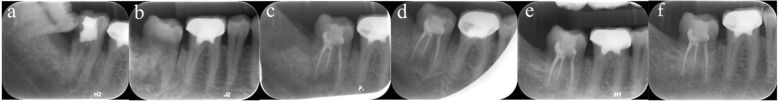
The X-ray photograph before surgery (**a**) and immediate (**b**), 1 (**c**), 3 (**d**), 12 (**e**), 24 (**f**) months after surgery

All the patients met the criteria of the success as we enumerated the points previously, and no periodontal pocket, mobility, inflammation and absorption of the root were found. No mobility was found in any cases during the follow-up period and only one patient felt slight pain from percussion of the transplanted tooth at 4 weeks. In addition, only one patient felt slight sensitivity when chewing soft food at 4 weeks. In terms of probing depth, three patients’ probing depth was deeper than 3 mm, and one patient’s probing depth was deeper than 4 mm at 4 weeks, all the probing sites were distal-buccal/lingual. Meanwhile, the probing depth at the distal-buccal/lingual site was deeper than 3 mm in one patient at 3-months follow-up whose probing depth was deeper than 4 mm at 4 weeks. The probing depths in other transplanted teeth were normal at all follow-ups. The specific data about the related clinical symptoms appear in Table 2. In terms of the X-rays, no sign of bone loss of more than one third of the root length, ankyloses, or root resorption occurred during the 2-year follow-up, as shown in Fig. 4.

**Table 2:** Number of patients who had the clinical symptom

Follow-up	mobility	Pain on percussion	No masticatory function	Probing depth 3 mm	Probing depth 4 mm
1 week	0	0	/	/	/
2 weeks	0	0	/	/	/
4 weeks	0	1	1 (slightly sensitive)	3 (distal-buccal/lingual)	1(distal-buccal/lingual)
3 months	0	0	0	1(distal-buccal/lingual)	0
6 months	0	0	0	0	0
12 months	0	0	0	0	0
24 months	0	0	0	0	0

## Discussions

In our retrospective study, the tooth autotransplantation, using 3D replica with or without GBR, was an efficient method with a 100% success rate during two-years follow-up, according the success criteria previously mentioned. As EzEldeen reported that the CBCT-guided tooth autotransplantation could be adopted as an alternative for the conventional approach with the help of 3D analysis [20]. Verweij et al. [17] also reported that high success rates were achieved when using donor tooth replicas, the success and survival rates of 80.0–91.1% and 95.5–100%, respectively. Healthy periodontal ligament and the good tissue adaptation are considered as the most important factors in successful tooth transplantation [15]. Meanwhile, the extraoral time, number of fitting attempts, skill of surgeon, and the trauma of the recipient socket may affect the periodontal ligament.

In the present study, we used a 3D model of donor tooth as a replica to prepared the recipient socket in order to preserve the periodontal ligament of the donor tooth. Firstly, the 3D replica of donor tooth can replace the real one to determine whether the recipient socket is ideally suited for the donor tooth which process would damage the periodontal ligament seriously. Second, the use of the 3D replica of donor tooth shorten the extraoral time to 0–4 min in our surgery. Meanwhile the use of minimally invasive technique can reduce the damage of the periodontal ligament during the extraction of the donor tooth. Andreasen et al. reported that the normal periodontal healing would proceed if the extraoral time of the donor tooth was less than 18 min [11]. The extraoral time in our cases were much less than 18 min and were consistent with other clinical studies. As Shahbazian et al. reported that the average extra-alveolar time was < 1 min for the 3D replica group and up to 3–10 min in the conventional group [19].

In our cases, there are two cases cost 3.5 and 4 min due to the error range of the 3D replica, that is the inaccuracy of the model. The accuracy of the 3D replica model is important to the process of the surgery. The accuracy of the 3D replica model also effected the fitness of the donor teeth to the recipient socket. Many factors may affect the accuracy of the replica model, such as the data from the CBCT, the material shrinkage during the building or postcuring and the minimal thickness of the layers [24]. So far there is no standard definition of the clinically acceptable differences between the replica model and the donor teeth, although several studies reported that the difference of less than 0.25 mm is clinically acceptable [25]. And Lee et al. reported that the mean deviations of the replica model manufactured by 3D printer were 0.038–0.047 mm [26], which is much less than the clinically acceptable value. Also Lee and Kim reported that the 3D replica models were, on an average, 0.149 mm smaller in size than the real teeth [27]. And Khalil et al. proved that the dimensional differences between the 3D replica models made by 3D printing technologies and the real teeth were below 0.25 mm, which is accepted by the clinical demand [28]. Therefore, the 3D printing technologies, used for 3D replica models of the donor teeth, is accuracy enough for the autotransplantation of the teeth. The fitness of the donor teeth to the recipient socket was well in our clinical operation, expect the two cases due to the date of the CBCT was incomplete during the date transmission and the inaccuracy of the segmentation of the donor tooth.

The use of 3D replica model of donor tooth can not only reduce the damage to the periodontal ligament but also increase the ease of the surgery of the tooth autotransplantation and decrease the requirement for the experience of the surgeon. Verweij et al. demonstrated that the surgery time of the autotransplantation when using replica model can be shorten to less than 30 min even if the surgery was done by a less experienced surgeon [29]. Shahbazian et al. compared the traditional technique to 3D autotransplantation and found that the time of the surgery proceduce were 40–90 min and 30–45 min, respectively [19].

Many other factors affect the success of the autotransplantation tooth. Yoshino et al. analyzed the influence of age on the tooth autotransplantation and found that the younger the patient is, the higher success rate of the tooth autotransplantation, the success rate was lower in the 55–69 years old group [30]. Sugai et al. and Yoshino et al. also reported that patients under 40 years old showed a higher success rate than the older one group [31, 32]. Yoshino et al. also analyzed the influence of gender on the tooth autotransplantation and found that the survival rate of the tooth autotransplantation of males was lower at 5-years, 10-years and 15-years follow-ups and need more attention during the autotransplantation process compared with female [33]. Therefore, the use of donor tooth replica is more needed in male patients so that the surgery process can be handle well.

The third molars extracted for autotransplantation in all cases in the present study were mature teeth with developed roots, so the revascularization of the pulp is not likely to happen after transplantation and needed root canal therapy [31, 34]. Some cases in the present study use the GBR to regeneration the bone defect or fill the gap when the recipient site was too large for the donor tooth. Bone defects often occur around the compromised tooth, and a lack of buccal bone has been reported to contribute to the failure of the tooth autotransplantation [35]. When the fresh extraction socket is too large for the donor tooth, the extra space for transplant placement is generally filled by the autogenous bone. The exposure of the root surface may reduce the success rate of the PDL healing [21, 36]. The bone defect around the transplanted tooth may affect the regeneration of new cementum and periodontal of the root surface, which may course periodontal disease and a bad prognosis [37, 38]. Compared with xenogenic bone, autogenous bone that was collected from extraction socket has the capable of osteogenesis, osteoinduction, and osteoconduction, and may reduce the forgein-body reaction [39]. Yu. HJ et al. reported that using GBR during autotransplantation in recipient site where buccolingual alveolar bone atrophy could also result in a good long-term outcome [22]. Other studies also proved that the usefulness of GBR in the autotransplantation at recipient sites with bone defects [21]. The success rate of using GBR in autotransplantation is consistent with the non GBR one in the present study.

The success rate of the autotransplantation, using 3D replica, is high, but the long-term survival rate still need to be observed, and the precise of the tooth autotransplantation need not only a 3D replica as a guide but also a preparation guide of the recipient site and a guide for occlusion, all of which still need more research.

## Conclusion

The tooth autotransplantation using 3D replica with or without GBR is an effective method which can reduce the extra-oral time of the donor teeth and may result in less failure. And the new 3D replica of donor tooth can make the surgery of tooth autotransplantation much easier and faster.

## Data Availability

The datasets used and/or analysed during the current study are available from the corresponding author on reasonable request.

## Abbreviations

3D: Three Dimensions
CBCT: Cone-beam computed tomography
CGFs: Concentrated growth factors
GBR: Guided bone regeneration

## References

[1] Cross D, El-Angbawi A, McLaughlin P (2013). Developments in autotransplantation of teeth. Surgeon.

[2] Almpani K, Papageorgiou SN, Papadopoulos MA (2015). Autotransplantation of teeth in humans: a systematic review and meta-analysis. Clin Oral Investig.

[3] Verweij JP, Anssari Moin D, Mensink G (2016). Autotransplantation 2.0. Considerations, results and the latest techniques. Ned Tijdschr Tandheelkd.

[4] Ong D, Itskovich Y, Dance G (2016). Autotransplantation: a viable treatment option for adolescent patients with significantly compromised teeth. Aust Dent J.

[5] Czochrowska EM, Stenvik A, Album B (2000). Autotransplantation of premolars to replace maxillary incisors: a comparison with natural incisors. Am J Orthod Dentofacial Orthop.

[6] Jang JH, Lee SJ, Kim E (2013). Autotransplantation of immature third molars using a computer-aided rapid prototyping model: a report of 4 cases. J Endod.

[7] Cardona JL, Caldera MM, Vera J (2012). Autotransplantation of a premolar: a long-term follow-up report of a clinical case. J Endod.

[8] Cousley RRJ, Gibbons A, Nayler J (2017). A 3D printed surgical analogue to reduce donor tooth trauma during autotransplantation. J Orthod.

[9] Rohof ECM, Kerdijk W, Jansma J (2018). Autotransplantation of teeth with incomplete root formation: a systematic review and meta-analysis. Clin Oral Investig.

[10] Ronchetti MF, Valdec S, Pandis N (2015). A retrospective analysis of factors influencing the success of autotransplanted posterior teeth. Prog Orthod.

[11] Andreasen JO, Paulsen HU, Yu Z (1990). A long-term study of 370 autotransplanted premolars. Part II. Tooth survival and pulp healing subsequent to transplantation. Eur J Orthod.

[12] Andreasen JO, Paulsen HU, Yu Z (1990). A long-term study of 370 autotransplanted premolars. Part IV. Root development subsequent to transplantation. Eur J Orthod.

[13] Andreasen JO, Paulsen HU, Yu Z (1990). A long-term study of 370 autotransplanted premolars. Part I. Surgical procedures and standardized techniques for monitoring healing. Eur J Orthod.

[14] Denys D, Shahbazian M, Jacobs R (2013). Importance of root development in autotransplantations: a retrospective study of 137 teeth with a follow-up period varying from 1 week to 14 years. Eur J Orthod.

[15] Kim E, Jung JY, Cha IH (2005). Evaluation of the prognosis and causes of failure in 182 cases of autogenous tooth transplantation. Oral Surg Oral Med Oral Pathol Oral Radiol Endod.

[16] Lee SJ, Jung IY, Lee CY (2001). Clinical application of computer-aided rapid prototyping for tooth transplantation. Dent Traumatol.

[17] Verweij JP, Jongkees FA, Anssari Moin D (2017). Autotransplantation of teeth using computer-aided rapid prototyping of a three-dimensional replica of the donor tooth: a systematic literature review. Int J Oral Maxillofac Surg.

[18] Lee SJ (2004). Clinical application of computer-aided rapid prototyping for tooth transplantation. Aust Endod J.

[19] Shahbazian M, Jacobs R, Wyatt J (2013). Validation of the cone beam computed tomography-based stereolithographic surgical guide aiding autotransplantation of teeth: clinical case-control study. Oral Surg Oral Med Oral Pathol Oral Radiol.

[20] EzEldeen M, Wyatt J, Al-Rimawi A (2019). Use of CBCT guidance for tooth autotransplantation in children. J Dent Res.

[21] Yu HJ, Qiu LX, Wang XZ (2014). Long-term follow-up of autogenous canine transplants with application of guided bone regeneration. Int J Oral Maxillofac Surg.

[22] Yu HJ, Jia P, Lv Z (2017). Autotransplantation of third molars with completely formed roots into surgically created sockets and fresh extraction sockets: a 10-year comparative study. Int J Oral Maxillofac Surg.

[23] Tsukiboshi MAJ, Asai Y (2001). Autotransplantation of teeth.

[24] Barker TM, Earwaker WJ, Lisle DA (1994). Accuracy of stereolithographic models of human anatomy. Australas Radiol.

[25] Hazeveld A, Huddleston Slater JJ, Ren Y (2014). Accuracy and reproducibility of dental replica models reconstructed by different rapid prototyping techniques. Am J Orthod Dentofacial Orthop.

[26] Lee KY, Cho JW, Chang NY (2015). Accuracy of three-dimensional printing for manufacturing replica teeth. Korean J Orthod.

[27] Lee SJ, Kim E (2012). Minimizing the extra-oral time in autogeneous tooth transplantation: use of computer-aided rapid prototyping (CARP) as a duplicate model tooth. Restor Dent Endod.

[28] Khalil W, EzEldeen M, Van De Casteele E (2016). Validation of cone beam computed tomography-based tooth printing using different three-dimensional printing technologies. Oral Surg Oral Med Oral Pathol Oral Radiol.

[29] Verweij JP, Moin DA, Mensink G (2016). Autotransplantation of premolars with a 3-dimensional printed titanium replica of the donor tooth functioning as a surgical guide: proof of concept. J Oral Maxillofac Surg.

[30] Yoshino K, Kariya N, Namura D (2013). Influence of age on tooth autotransplantation with complete root formation. J Oral Rehabil.

[31] Sugai T, Yoshizawa M, Kobayashi T (2010). Clinical study on prognostic factors for autotransplantation of teeth with complete root formation. Int J Oral Maxillofac Surg.

[32] Yoshino K, Kariya N, Namura D (2012). Risk factors affecting third molar autotransplantation in males: a retrospective survey in dental clinics. J Oral Rehabil.

[33] Yoshino K, Ishizuka Y, Sugihara N (2013). Gender difference in tooth autotransplantation with complete root formation: a retrospective survey. J Oral Rehabil.

[34] Mejare B, Wannfors K, Jansson L (2004). A prospective study on transplantation of third molars with complete root formation. Oral Surg Oral Med Oral Pathol Oral Radiol Endod.

[35] Ko JM, Paik CH, Choi S (2014). A patient with protrusion and multiple missing teeth treated with autotransplantation and space closure. Angle Orthod.

[36] Imazato S, Fukunishi K (2004). Potential efficacy of GTR and autogenous bone graft for autotransplantation to recipient sites with osseous defects: evaluation by re-entry procedure. Dent Traumatol.

[37] Jang Y, Choi YJ, Lee SJ (2016). Prognostic factors for clinical outcomes in autotransplantation of teeth with complete root formation: survival analysis for up to 12 years. J Endod.

[38] Aoyama S, Yoshizawa M, Niimi K (2012). Prognostic factors for autotransplantation of teeth with complete root formation. Oral Surg Oral Med Oral Pathol Oral Radiol.

[39] Annunziata Marco, Piccirillo Angelantonio, Perillo Francesco, Cecoro Gennaro, Nastri Livia, Guida Luigi (2019). Enamel Matrix Derivative and Autogenous Bone Graft for Periodontal Regeneration of Intrabony Defects in Humans: A Systematic Review and Meta-Analysis. Materials.

